# Simvastatin Impairs the Inflammatory and Repair Phases of the Postinjury Skeletal Muscle Regeneration

**DOI:** 10.1155/2018/7617312

**Published:** 2018-11-04

**Authors:** Iwona Otrocka-Domagała, Katarzyna Paździor-Czapula, Tomasz Maślanka

**Affiliations:** ^1^Department of Pathological Anatomy, Faculty of Veterinary Medicine, University of Warmia and Mazury, Oczapowskiego Street 13, 10-719 Olsztyn, Poland; ^2^Department of Pharmacology and Toxicology, Faculty of Veterinary Medicine, University of Warmia and Mazury, Oczapowskiego Street 13, 10-719 Olsztyn, Poland

## Abstract

**Background:**

Recent clinical data have suggested that the chronic use of high-lipophilic statins impairs the regenerative capacity of skeletal muscle. Because this activity of statins is poorly understood, we aimed to investigate the effect of simvastatin (SIM) on postinjury myofibre regeneration.

**Methods:**

The porcine model was used in this study. The animals were divided into two groups: nontreated (control;* n*=24) and SIM-treated (40 mg/day;* n*=24). On the 15th day (day 0) of the experiment, a bupivacaine hydrochloride- (BPVC-) induced muscle injury was established, and the animals were sacrificed in the following days after muscle injury. The degree of regeneration was assessed based on histopathological and immunohistochemical examinations. The presence and degree of extravasation, necrosis, and inflammation in the inflammatory phase were assessed, whereas the repair phase was evaluated based on the numbers of muscle precursor cells (MPCs), myotube and young myofibres.

**Results:**

In the inflammatory phase, SIM increased the distribution and prolonged the period of extravasation, prolonged the duration of necrosis, and prolonged and enhanced the infiltration of inflammatory cells. In the repair phase, SIM delayed and prolonged the activity of MPCs, delayed myotube formation, and delayed and decreased the formation of young myofibres. Our results indicated that SIM did not improve blood vessel stabilization at the site of the injury, did not exert an anti-inflammatory effect, prolonged and enhanced the inflammatory response, and impaired MPC activity, differentiation, and fusion. Moreover, SIM appeared to reduce M1 macrophage activity, resulting in slower removal of necrotic debris and sustained necrosis.

**Conclusion:**

This study shows that SIM negatively affects the inflammatory and repair phases of the postinjury muscle regeneration. These findings are unique, strengthen the available knowledge on the side effects of SIM, and provide evidence showing that statin therapy is associated with an increased risk of impairment of the regenerative capacity of muscle.

## 1. Introduction

Postinjury skeletal muscle regeneration is a complex process, and its success depends on the precise control of a sequence of events. The initial inflammatory phase, which is characterized by myofibre necrosis, extravasation, and neutrophil infiltration and is rapidly followed by macrophage recruitment and phagocytosis of the myofibre debris, is very important for muscle reconstruction. During this phase, inflammatory cells interact with each other, and neutrophils are the first cell population found in acutely injured muscle, which peaked at 12-24 h postinjury. These cells can promote the cytolytic and phagocytic capacity of the M1 macrophage population, which reaches a peak at 2 days after injury. Furthermore, M1 macrophages are replaced by a population of nonphagocytic M2 macrophages, which reached a peak at 4 days after injury and can attenuate the inflammatory phase [1]. Subsequently, a repair phase, which is characterized by the activation, migration, proliferation, and differentiation of muscle precursor cells (MPCs; this cell population includes satellite cells and myoblasts) and myotube and young muscle fibre formation, is closely linked to the first phase at the cellular and molecular levels [1, 2]. It has been shown that M2 macrophages are crucial for the recruitment, proliferation, and differentiation of MPCs and for the promotion of the repair phase, and M1 macrophages also regulate the proliferation and early differentiation of MPCs [1, 3]. Intriguingly, MPCs themselves attract macrophages to the site of injury and use them as a support to escape apoptosis, particularly during their fusion [4, 5]. An important regulatory role also is also played by Treg cells, which reach a peak at 4 days after injury; these cells not only promote the transition of the macrophage phenotype from M1 to M2 but also stimulate MPCs and muscle regeneration [1, 6]. Eosinophils are equally important because they stimulate MPC differentiation [1, 6]. Therefore, the correct balance of cell populations and the accurate synchronization of the inflammatory and repair phases are essential for the proper course of the postinjury myofibre regeneration.

Despite their cholesterol-lowering effect, statins, as 3-hydroxy-3-methylglutaryl-coenzyme A (HMG-CoA) reductase inhibitors, also have cholesterol-independent (pleiotropic) actions, such as improving endothelial function, decreasing the generation of ROS, and attenuating inflammatory cell recruitment [7, 8]. Studies on the effects of simvastatin (SIM) pretreatment on cardiopulmonary bypass surgery outcomes have confirmed the beneficial effect of SIM on reducing the plasma concentrations of proinflammatory cytokines and increasing neutrophil apoptosis, resulting in less tissue damage after surgery [9]. Similarly, SIM affects macrophages through attenuation of the expression of several proinflammatory cytokines, factors, adhesion molecules, and molecules mediating inflammatory signalling and of the upregulation of Kruppel-like factor-2 (KLF-2) [10]. Recently, antithrombotic statin action has been considered [11–13]. This abovementioned statin property indicates that SIM can modulate the course of the inflammatory phase of the postinjury myofibre regeneration.

Recent studies have revealed that statins also exert adverse myotoxic effects resulting from membrane hyperexcitability, impairment of mitochondrial function, Ca^2+^ homeostasis, and ubiquinone depletion, as well as subsequent mitochondrial membrane depolarization and apoptosis in skeletal muscle [7, 14, 15]. Moreover, due to its lipophilic nature, which enable it to enter muscle cells and alter membrane structures, SIM has been associated with a greater risk of myopathy than other statins [7, 16]. Furthermore, some studies have suggested that SIM can impair the proliferation and differentiation of MPCs and reduce their fusion into myotubes [17–19]. Therefore, it should be assumed that SIM administration can mainly affect the repair phase of myofibre regeneration. Based on these statin properties and the fact that the long-term patients treated with SIM are mostly over 60 (men) or 65 (women) years of age, there is a possibility that SIM treatments could be a risk factor for age-related loss of skeletal muscle function and regenerative capacity [20, 21].

Due to the beneficial and adverse effects of statins, as well as the significant gap in knowledge regarding their impact on the course of striated myofibre regeneration, we decided to conduct an* in vivo* study in this research area. The results of the study might facilitate the assessment of risk factors during SIM therapy, which should particularly be taken into consideration during the treatment of elderly patients. The principal hypothesis presumed that SIM affects not only the repair phase but also the inflammatory phase of the regeneration. Furthermore, we hypothesized that the following SIM properties underlie this action: (a) improvement of endothelial function, (b) anti-inflammatory effects, (c) modulation of MPC proliferation and differentiation, and (d) myotoxicity. Based on these considerations, this study aimed to determine the effect of SIM treatment on the course of the inflammatory and repair phases of the skeletal muscle regeneration following experimental injury.

## 2. Materials and Methods

### 2.1. Animals and Study Design

**Figure 1:**
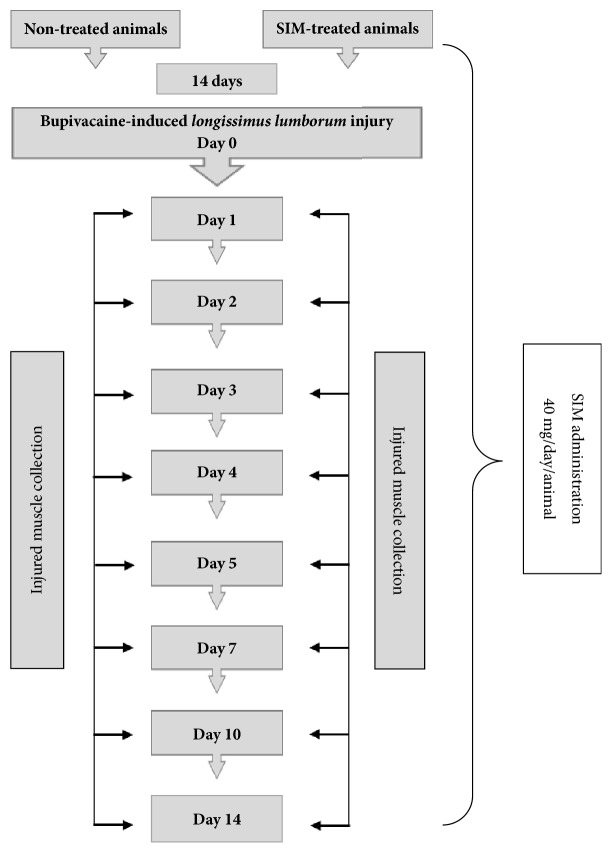
Scheme of experimental study design. The animals were divided into the nontreated (control) and SIM-treated groups. The oral administration of SIM (40 mg/day/animal) was started 14 days prior to muscle injury and was continued after injury. On the 15th day of the experiment (day 0), muscle injury was induced by BPVC. The animals were sacrificed at various days after the injury was induced (three gilts/group/experimental day), and muscle samples were collected for evaluation.

The experimental procedures utilized in this study were in accordance with governmental guidelines on animal experimentation and were approved by the Local Ethics Commission for Animal Experiments of Warmia and Mazury University in Olsztyn, Olsztyn, Poland (Decision No. 62/2010). The experiment was performed using 48 clinically healthy gilts (Polish large white breed) aged 3 months (at the start of the experiment) that originated from a large pig farm and were maintained indoors at the experimental section of the Faculty of Veterinary Medicine of Warmia and Mazury University in Olsztyn. Specifically, the animals were kept in ventilated 10 m^2^ pens (24 gilts per pen) on a concrete floor with rubber mat areas and a natural light/dark cycle and cleaned twice per day. In addition, the gilts were fed commercial grower feed twice per day and provided fresh water* ad libitum*. The piglets were maintained at 25°C during the 10-day acclimatization period before the start of the experiment and then at 23°C during the experiment. The animals were randomly divided into two experimental groups: group I (control) included 24 nontreated gilts and group II included 24 gilts that were treated* per os* with SIM (Simvasterol, Polpharma, Poland) at a daily dose of 40 mg per animal (approximately 1 mg/kg) from the first to the last day of the experiment. The dosage of SIM was selected based on published reports that indicated the low risk of myotoxicity observed with this dose [22, 23]. On the 15th day (day 0) of the experiment, two muscle injuries were induced through 10 ml injections of 0.5% bupivacaine hydrochloride (BPVC) solution (Marcaine, AstraZeneca, UK) into the right and left* longissimus lumborum* muscles (two independent injuries were induced in each animal, one was induced on the right* longissimus lumborum* muscle, and the other was induced the left* longissimus lumborum* muscle). The skin at the injection site was topically anaesthetized with 10% lidocaine (Lidocaine Spray, Egis, Budapest, Hungary) and marked with tattoo ink. The induction of muscle injury was preceded (20 min) by premedication with 2 mg/kg azaperone (Stresnil, Janssen Pharmaceutica NV, Beerse, Belgium) administered intramuscularly (i.m.) and 0.05 mg/kg atropine (Atropinum Sulfuricum, Polfa S.A, Warsaw, Poland) administered i.m. The animals were euthanized through the intravenous injection (i.v.) of 0.25 ml/kg of 40% pentobarbital sodium salt (Euthaminal, Alfasan, Nederland B.V) on days 1, 2, 3, 4, 5, 7, 10, and 14 after the induction of muscle injury (three gilts/per group/per time point). Twenty minutes before euthanasia, the gilts were premedicated with 2 mg/kg azaperone (Stresnil, Janssen Pharmaceutica NV, Beerse, Belgium) administered i.m. The experimental study design scheme is shown in Figure 1.

### 2.2. Microscopic Evaluation

Immediately after euthanasia, muscle samples from the injured sites at the right and left* longissimus lumborum* muscle (two longitudinal and two transverse sections of each site) were collected from each animal in both groups on days 1, 2, 3, 4, 5, 7, 10, and 14 after BPVC injection. The samples were fixed in neutralized 10% formalin, embedded in paraffin wax, and cut into 3 *μ*m thick sections. All longitudinal and transverse muscle sections were stained with haematoxylin (Mayer's; Sigma-Aldrich) and eosin (Sigma-Aldrich) (H&E) for histopathological examination (evaluation of extravasation, necrosis, inflammation, MPCs, myotubes, and young myofibres). The presence of MPCs was confirmed by MyoD1 and desmin expression and that of myotubes and young myofibres was confirmed by desmin expression, using the antibodies listed in Table 1. Immunohistochemical labelling was performed through an immunoperoxidase method using 3,3′-diamonobenzidine (DAB) as the chromogen (Table 1). Subsequently, the sections were counterstained with haematoxylin (Mayer's; Sigma-Aldrich). Positive and negative controls were processed with the evaluated slides in a similar manner.

The regeneration of myofibres was evaluated based on the presence of injury (extravasation, necrosis, and inflammation) and recovery (presence of MPCs, myotubes, and young myofibres) features assessed at 400x magnification in 10 fields of areas of muscular injury in each section (two longitudinal and two transverse sections). The presence of MPCs, myotubes, and young myofibres was confirmed by positive immunohistochemical labelling for MyoD1 (MPCs) and desmin (MPCs, myotubes and young myofibres) expression, and their numbers were assessed as the number of positive cells at 400x magnification in 10 fields of areas of muscular injury in each section (two longitudinal and two transverse sections). The scoring system used to evaluate the inflammatory and repair phases of BPVC-induced skeletal muscle regeneration was designed by the authors based on their experience with observations of the myofibre regeneration process (Table 2) [24, 25].

### 2.3. Statistical Analysis

The data are expressed as the mean (±SD) scores per injured muscle area. The data represent information from six sites of injured muscle per group per day [three animals and two independent muscle injuries per animal per day]. The variables were checked for normality using the Shapiro-Wilk test. Student's unpaired *t*-test was used to compare the results between the SIM-treated and control groups. A statistical analysis of the dynamics (i.e., multiple comparisons between particular time points within a group) of injury and recovery features in the SIM-treated and control groups was performed using one-way analysis of variance (ANOVA) followed by Bonferroni's* post hoc *test. The differences were considered significant if the P values were less than 0.05. SigmaPlot Software Version 12.0 (Systat Software Inc., San Jose, CA, USA) was used for the statistical analyses and the preparation of graphs.

## 3. Results

### 3.1. SIM Increases the Distribution and Prolongs the Period of Extravasation in BPVC-Injured Muscles

**Figure 2:**
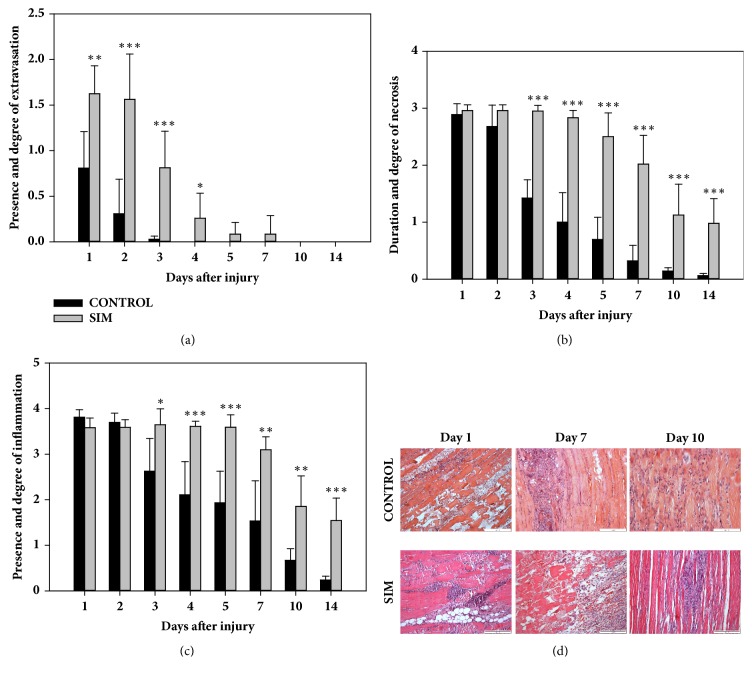
Effect of SIM on the inflammatory phase of the postinjury skeletal muscle regeneration. To assess this effect, the presence and degree of extravasation (a), the duration and degree of necrosis (b), and the presence and intensity of inflammation (c) were evaluated in the BPVC-injured muscles of nontreated (control) and SIM-treated animals. The results are expressed as the mean scores (±SDs) from six sites of muscle injury derived from three animals (two independent muscle injuries per single animal) per group per day. The P values (^*∗*^*P* < 0.05, ^*∗∗*^*P* < 0.01, and ^*∗∗∗*^*P* < 0.001) indicate the significance of the differences between groups at the same time point. Representative H&E-stained sections of postinjury myofibre regeneration sites. Day 1: extensive necrosis and marked inflammation were observed in the control and SIM-treated groups. Day 7: mild inflammation, moderately numerous myotubes and numerous young myofibres were observed in the control group. Extensive necrosis and extravasation, moderate inflammation, and moderately numerous myotubes were detected in the SIM-treated group. Day 10: not numerous myotubes and numerous young myofibres were found in the control group, whereas focal necrosis, mild inflammation, not numerous myotubes, and moderately numerous young myofibres were detected in the SIM-treated group (d).

**Figure 3:**
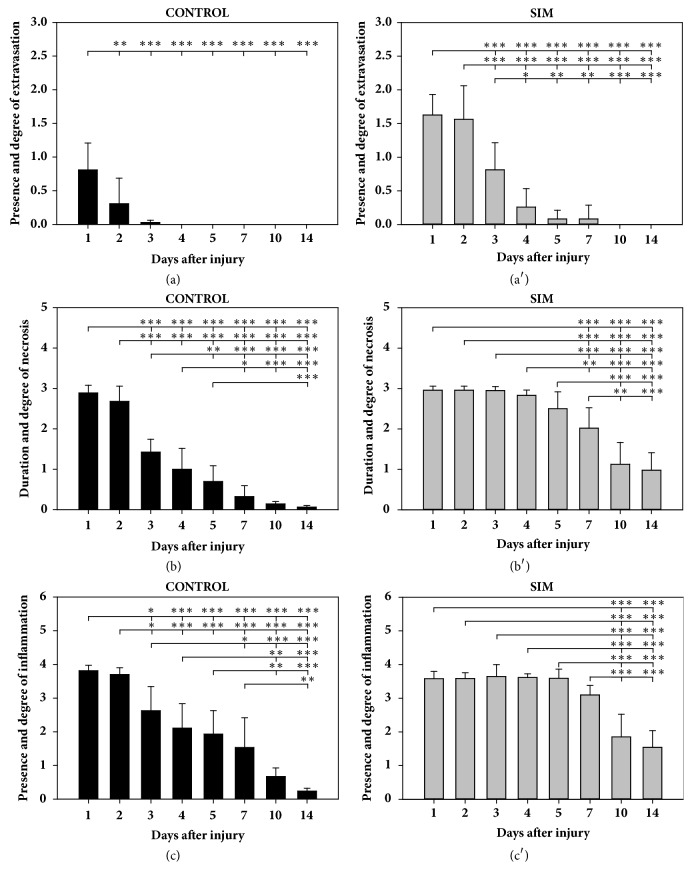
Effect of SIM on the dynamics of the inflammatory phase of the postinjury skeletal muscle regeneration. To assess this effect, the presence and degree of extravasation (a, a′), the duration and degree of necrosis (b, b′), and the presence and intensity of inflammation (c, c′) were compared between particular time points in the BPVC-injured muscles of nontreated (control) or SIM-treated animals. The results are expressed as the mean scores (±SDs) from six sites of muscle injury derived from three animals (two independent muscle injuries per single animal) per day in the control ((a), (b), and (c)) and SIM-treated ((a′), (b′), and (c′)) groups. The* P* values (^*∗*^*P* < 0.05, ^*∗∗*^*P* < 0.01, and ^*∗∗∗*^*P* < 0.001) indicate the significance of the differences between all the time points in the control group or SIM-treated group.

SIM resulted in significant increase the severity of extravasation on day 1 compared with the control group (Figure 2(a)). Similarly, on days 2, 3, and 4, significant increases (*P *< 0.001,* P *< 0.001, and* P *< 0.05, respectively; Figure 2(a)) in this parameter were observed in the SIM-treated animals compared with the control group. On days 5 and 7, statistically significant differences in the mean extravasation values were not observed between the groups; however, focal extravasations were still observed in the SIM-treated animals at these time points, while they were absent from day 4 in the control group (Figure 2(a)). On days 10 and 14, no extravasation was detected in either group. These results indicated that SIM increased the distribution and prolonged the period of extravasation in BPVC-injured muscles. A comparative analysis of the dynamics of this parameter in the SIM-treated and nontreated animals at all analysed time points confirmed this conclusion (Figures 3(a) and 3(a′)). The administration of SIM caused extensive extravasations on days 1 (1.62 ± 0.30) and 2 (1.56 ± 0.49) (Figure 3(a′)), and no significant differences were found between these time points. The extravasations were distributed focally on days 1 (0.80 ± 0.40) and 2 (0.30 ± 0.37) in the nontreated animals, and a significant decrease (*P* < 0.01) in this parameter was noted from day 1 to 2 (Figure 3(a)). On days 3 and 4, extravasations were considerably reduced in the SIM-treated group (0.81 ± 0.40 versus 0.25 ± 0.27; Figure 3(a′)), resulting in significant differences in the mean value of this parameter between days 2, 3, and 4 (*P *< 0.01,* P *< 0.05, respectively; Figure 3(a′)). In contrast, in the nontreated animals, the mean value of extravasations remained low on day 3 (0.02 ± 0.03), and no significant differences were detected between days 2 and 3 (Figure 3(a)). After SIM treatment, extravasations were still observed on days 5 and 7, and their distribution did not considerably decrease during this period (Figures 2(d) and 3(a′)).

### 3.2. SIM Prolongs the Duration of Necrosis in BPVC-Injured Muscles

SIM did not significantly increase the mean degrees of necrosis on days 1 and 2 compared with those in the control animals (Figures 2(b) and 2(d)). However, on days 3, 4, 5, 7, 10, and 14, the mean extension of necrosis was significantly higher in the treated animals than that in the control animals (day 3:* P* < 0.001; 4:* P* < 0.001; 5:* P* < 0.001; 7:* P* < 0.001; 10:* P* < 0.001; 14:* P* < 0.001; Figure 2(b)). These results indicated that SIM prolonged the duration of necrosis in BPVC-injured muscles. A comparative analysis of the dynamics of this parameter in the SIM-treated and nontreated animals at all analysed time points confirmed this conclusion (Figures 3(b) and 3(b′)). These findings demonstrated that necrosis was extensive on days 1 and 2 (2.95 ± 0.10 versus 2.95 ± 0.1; Figure 3(b′)) in the SIM-treated and nontreated (2.88 ± 0.19 versus 2.67 ± 0.37; Figure 3(b)) animals, without significant differences between the time points in either group. On day 3, this parameter was significantly decreased (*P* < 0.001) compared with that on day 2 only in the control animals. On day 4, a significant reduction in necrosis was not observed in either group compared with that on day 3; however, in the SIM-treated animals, necrosis remained extensive at this time point, whereas the control animals showed necrosis only in single fibres (Figures 3(b) and 3(b′)). On days 5 and 7, a significant reduction in this parameter was not detected in either the SIM-treated or control group; however, in the SIM-treated animals, necrosis remained extensively or focally distributed (day 5: 2.50 ± 0.41; 7: 2.02 ± 0.50; Figures 2(d) and 3(b′)), whereas, in the nontreated animals, necrosis was only detected in only single fibres (day 5: 0.69 ± 0.38; 7: 0.32 ± 0.27; Figure 3(b)). In the SIM-treated animals, necrosis was decreased significantly (*P* < 0.01) on day 10 compared with that on day 7 and remained focally distributed (Figure 2(d)) or limited to only single fibres (1.25 ± 0.54), and the mean degree of necrosis remained at a similar level on day 14 (0.97 ± 0.43), with no significant differences between days 10 and 14 (Figure 3(b′)). This result confirmed that the final level of necrosis was reached on day 10 in the SIM-treated group. In contrast, in the control animals, the mean extension of necrosis did not differ significantly between days 7, 10, and 14, and, at these time points, necrosis was restricted to single fibres or was not detected (Figure 3(b)), which indicated that the final level of necrosis was reached on day 7 in this group. These results demonstrated that SIM prolonged the duration of necrosis in BPVC-injured muscles by one time point compared with the control animals, irrespective of the severity of this process in the statin-treated animals.

### 3.3. SIM Prolongs and Enhances the Infiltration of Inflammatory Cells in BPVC-Injured Muscles

On days 1 and 2, the mean severity of inflammation did not significantly differ between the SIM-treated and the nontreated animals (Figure 2(c)). However, on days 3, 4, 5, 7, 10, and 14, the mean value of this parameter was significantly higher in the SIM-treated animals than in the control group (day 3:* P* < 0.05; 4:* P* < 0.001; 5:* P* < 0.001; 7:* P* < 0.01; 10:* P* < 0.01; 14:* P* < 0.001; Figure 2(c)). These results indicated that SIM prolonged and enhanced the infiltration of inflammatory cells in BPVC-injured muscles. A comparative analysis of the dynamics of the inflammation severity in the statin-treated and nontreated animals at all analysed time points supports this conclusion (Figures 3(c) and 3(c′)). On days 1 and 2, marked inflammation was detected in the SIM-treated and control groups (Figure 2(d)), and the mean value of this parameter did not differ significantly between these time points in either group (Figures 3(c) and 3(c′)). On day 1, neutrophils were predominantly observed in the BPVC-injured muscles in both groups, whereas, on day 2, the inflammatory cell population was dominated by macrophages and contained a smaller proportion of neutrophils. In the nontreated animals, the mean intensity of inflammation was significantly decreased (*P* < 0.05; Figure 3(c)) on day 3 to a moderate degree compared with that on day 2. In contrast, in the SIM-treated group, marked inflammation was still detected on day 3, and the mean value did not differ considerably compared with that on day 2 (Figure 3(c′)). In both groups, macrophages were predominant in the injured muscles on day 3, and neutrophils and lymphocytes were observed occasionally. On days 4, 5, and 7, the mean number of inflammatory cells in the nontreated animals systematically decreased (day 4: 2.10 ± 0.72; 5: 1.93 ± 0.69; 7: 1.53 ± 0.88; Figures 2(d) and 3(c)), with no significant differences between these time points; however, each of these values was considerably lower than those obtained on days 1 and 2. In the SIM-treated animals, the intensity of inflammation on days 4, 5, and 7 remained high to moderate (day 4: 3.61 ± 0.11; 5: 3.59 ± 0.27; 7: 3.09 ± 0.28; Figures 2(d) and 3(c′)), and no significant differences were found between these time points. Furthermore, each of these values was not considerably lower than those on days 1 and 2 (Figure 3(c′)). This analysis showed that SIM maintained the severity of inflammation at a high level from day 1 to 7, whereas, during this time period, the value of this parameter decreased systematically in the control animals. On day 10, the nontreated animals showed minimal or no inflammation, and the mean number of inflammatory cells did not differ considerably compared with that on day 7 (Figure 3(c)). Similarly, no significant difference in this parameter was observed between days 10 and 14 in the control group (Figure 3(c)). In contrast, in the statin-treated animals, the mean severity of inflammation was significantly decreased (*P* < 0.001) on day 10 compared with that on day 7, although it remained at a mild to minimal level (Figure 2(d)). In addition, a similar mild to minimal level was detected at the last time point, with no significant difference between days 10 and 14 (Figure 3(c′)). In both groups, the inflammatory cell populations from day 4 to the last analysed time point were dominated by macrophages, contained a lower proportion of lymphocytes, and occasionally included eosinophils.

### 3.4. SIM Delays and Prolongs the Activity of MPCs in BPVC-Injured Muscles

**Figure 4:**
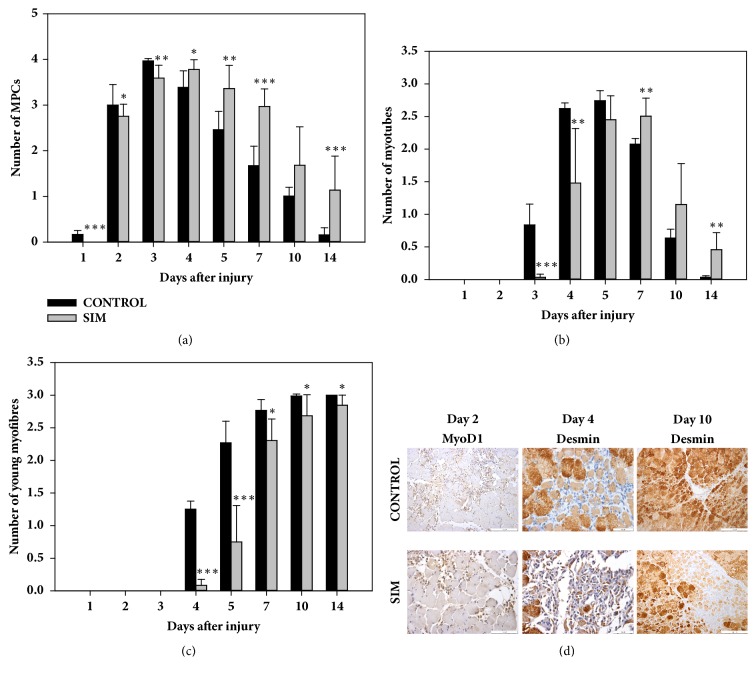
Effect of SIM on the repair phase of the postinjury skeletal muscle regeneration. To assess this effect, the mean numbers of muscle precursor cells (MPCs) (a), myotubes (b), and young myofibres (c) were evaluated in the BPVC-injured muscles of nontreated (control) and SIM-treated animals. The results represent the mean scores (±SDs) from six sites of muscle injury derived from three animals (two independent muscle injuries per single animal) per group per day. The* P* values (^*∗*^*P* < 0.05, ^*∗∗*^*P *< 0.01, and ^*∗∗∗*^*P* < 0.001) indicate the significance of the differences between the groups at the same time point. Representative immunohistochemistry of the myofibre regeneration sites after injury. Day 2: markedly numerous MyoD1-positive MPCs were detected in the control group, and numerous MyoD1-positive MPCs were found in the SIM-treated group. Day 4: numerous desmin-positive myotubes and not numerous young myofibres were found in the control group, and numerous desmin-positive MPCs and not numerous myotubes were detected in the SIM-treated group. Day 10: numerous desmin-positive young myofibres were observed in the control group. Not numerous desmin-positive myotubes and numerous young myofibres were found in the SIM-treated group. All sections were counterstained with haematoxylin (d).

**Figure 5:**
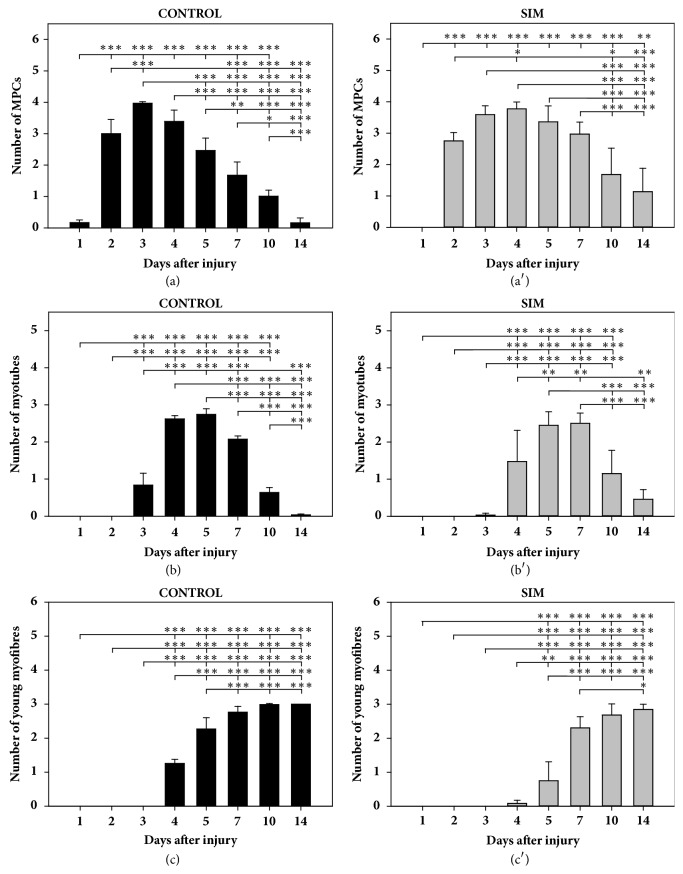
Effect of SIM on the dynamics of the repair phase of the postinjury skeletal muscle regeneration. To assess this effect, the mean numbers of muscle precursor cells (MPCs) (a, a′), myotubes (b, b′), and young myofibres (c, c′) were compared between particular time points in the BPVC-injured muscles of nontreated (control) or SIM-treated animals. The results are expressed as the mean scores (±SDs) from six sites of muscle injury derived from three animals (two independent muscle injuries per single animal) per day in the control ((a), (b), and (c)) and SIM-treated ((a′), (b′), and (c′)) groups. The* P* values (^*∗*^*P* < 0.05, ^*∗∗*^*P* < 0.01, and ^*∗∗∗*^*P* < 0.001) indicate the significance of the differences between all the time points in the control group or SIM-treated group.

The presence of MPCs was evaluated by H&E staining and confirmed by the expression of MyoD1 and desmin, and the results showed that SIM delayed the activation of MPCs. On day 1, MPCs were not detected, and a significant difference (*P* < 0.001) in the mean MPC number was found between the SIM-treated and nontreated animals (Figure 4(a)). On days 2 and 3, the number of MPCs in the statin-treated animals was also considerably lower (day 2:* P* < 0.05; 3:* P* < 0.01) than the corresponding control values (Figure 4(a)). In contrast, on days 4, 5, and 7, the numbers of MPCs were significantly increased (day 4:* P* < 0.05; 5:* P* < 0.01; 7:* P* < 0.001; Figure 4(a)) in the SIM-treated animals compared with those in the control group. On day 10, significant differences in this parameter were not found between the groups; nevertheless, the mean MPC number was distinctly higher (1.68 ± 0.84) in the SIM-treated animals than in the nontreated animals (1.00 ± 0.19) (Figure 4(a)). In contrast, at the last time point, the numbers of MPCs were significantly increased (*P* < 0.001) in the SIM-treated group than in the control animals (Figure 4(a)). This comparative analysis of the mean numbers of MPCs between the groups indicated that SIM delayed and prolonged the activity of MPCs in BPVC-injured muscles. This conclusion was also confirmed by an analysis of the variability of this parameter in the SIM-treated and control groups (Figures 5(a) and 5(a′)). In the statin-treated animals, no MPCs were detected on day 1, and compared with that on day 1, a considerably higher number of MPCs (*P* < 0.001; Figure 5(a′)) was observed on day 2, resulting in a moderately numerous or numerous MPCs (Figure 4(d)). In the nontreated animals on day 1, MPCs were either not observed or not numerous, whereas, on day 2, numerous MPCs were detected (Figure 4(d)), and their number was significantly increased (*P* < 0.001; Figure 5(a)) compared with that on day 1. In the SIM-treated animals, the number of MPCs on day 3 was equal to that at day 2 (Figure 5(a′)). In the nontreated animals, this parameter was considerably higher (*P* < 0.001; Figure 5(a)) on day 3 than on day 2 and reached the highest value noted throughout the entire experiment. In the SIM-treated group, the MPC number was not considerably increased on day 4 compared with that on day 3, and MPCs were numerous or markedly numerous (Figures 4(d) and 5(a′)). In contrast, no significant decrease in this parameter was noted between days 3 and 4 in the control animals (Figure 5(a)). In the treated animals, the MPC value did not differ significantly between days 4 and 5 (Figure 5(a′)), whereas, in the nontreated animals, the MPC number was significantly decreased (*P* < 0.001; Figure 5(a)) on day 5 compared with that on day 4, and further systematic significant decreases in this parameter were noted from day 5 to days 7, 10, and 14 (*P* < 0.01,* P* < 0.05, and* P* < 0.001, respectively; Figure 5(a)). In the SIM-treated animals, the number MPCs on day 7 was not considerably decreased compared with that on day 5; however, a significant decrease (*P* < 0.001) in their number was found on day 10 compared with that on day 7 (Figure 5(a′)). At the last analysed time point, no significant depletion of the mean MPC number compared with that on day 10 was noted in the SIM-treated animals, and not numerous MPCs were still observed (Figure 5(a′)).

### 3.5. SIM Delays Myotube Formation in BPVC-Injured Muscles

Myoblast fusion associated with the formation of multinucleated, small-diameter, desmin-positive myotubes started on day 3 in the nontreated and SIM-treated animals. However, in the SIM-treated animals, the myotube number was significantly reduced (day 3:* P* < 0.001; 4:* P* < 0.01) on days 3 and 4 compared with the control values (Figure 4(b)). On day 5, statistically significant differences in this parameter were not found between the groups; nevertheless, on days 7 and 14, but not day 10, the mean myotube number was considerably increased (day 7: P < 0.01; day 14: P < 0.01; Figure 4(b)) in the SIM-treated animals compared with the control values. These results indicated that SIM delayed myotube formation and prolonged their presence in the repair phase of BPVC-injured muscle regeneration. Comparative analyses of the dynamics of this parameter performed separately in the SIM-treated and nontreated animals confirmed this conclusion (Figures 5(b) and 5(b′)). In the SIM-treated animals, either no myotubes or single myotubes were observed on day 3; however, compared with that on day 3, their number was significantly increased (*P *< 0.001; Figure 5(b′)) on day 4, resulting in a nonnumerous or moderately numerous population (Figure 4(d)). In the control group, this parameter also differed significantly (*P *< 0.001; Figure 5(b)) between days 3 and 4, and myotubes were moderately numerous or numerous (Figure 4(d)) on day 4. After statin treatment, the myotube number was considerably increased (*P *< 0.01; Figure 5(b′)) on day 5 compared with that on day 4, whereas this value did not differ significantly between these time points in the control animals. In contrast, on day 7, the mean myotube number in the nontreated animals was significantly decreased (*P *< 0.001) compared with that on day 5, and myotubes were moderately numerous (Figure 2(d)). In addition, a significant decrease in this parameter was also noted from day 7 to days 10 and 14 (*P *< 0.001 and* P *< 0.001, respectively; Figures 2(d) and 5(b)). Although the SIM-treated animals did not show a significant difference in the myotube number between days 5 and 7, they were moderately numerous or numerous (Figure 2(d)). However, a significant decrease (*P *< 0.001) in this parameter was found on day 10 compared with that on day 7 (Figures 2(d), 4(d), and 5(b′)), but this decrease in the mean myotube number from day 10 to 14 was not significant, and a nonnumerous number of myotubes was still observed in these animals (Figure 5(b′)).

### 3.6. SIM Delays and Decreases Young Myofibre Formation in BPVC-Injured Muscles

Young myofibres with initially centrally located nuclei and a basophilic, desmin-positive cytoplasm showing a variable sarcomeric pattern were observed on day 4 in the nontreated (Figure 4(d)) and SIM-treated animals. From the beginning of the experiment until day 14, their number increased progressively in both groups. However, in the animals treated with SIM, the mean number of young myofibres was significantly reduced compared with that in the control group at all analysed time points (day 4:* P* < 0.001; 5:* P* < 0.001; 7:* P* < 0.05; 10:* P* < 0.05; 14:* P* < 0.05; Figure 4(c)). These results indicated that SIM delayed and decreases the formation of young myofibres. A comparative analysis of the dynamics of this parameter at all analysed time points in both groups also confirmed that young myofibre formation was slower in the SIM-treated animals compared with the control animals (Figures 5(c) and 5(c′)). This result was demonstrated by the finding that the final level of young myofibres in the nontreated animals was achieved on day 7 (Figure 2(d)), and this parameter did not differ significantly between days 7, 10, and 14 (Figures 4(d) and 5(c)). In the statin-treated animals, the value of this parameter was considerably lower (*P* < 0.05) on day 7 compared with that on day 14 and did not differ significantly between days 7 and 10 (Figures 2(d), 4(d), and 5(c′)), which indicated that the muscle fibre regeneration process was not completed by day 10 in this group.

## 4. Discussion

The results of the present study provide evidence for and support the principal hypothesis that SIM affects both the inflammatory and repair phases of skeletal muscle regeneration process after BPVC-induced injury. However, the effects of SIM on both regeneration phases were negative, resulting in a prolonged repair process compared with that observed in the control group. In the inflammatory phase, statin increased the distribution and prolonged the period of extravasation, prolonged the duration of necrosis, and prolonged and enhanced the infiltration of inflammatory cells. In the repair phase, SIM delayed and prolonged MPC activity, delayed myotube formation, and delayed and decreased young myofibre formation. These results indicated that statin did not improve blood vessel stabilization at the site of the injury, did not exert an anti-inflammatory effect, caused a prolonged increase in the inflammatory cell number, impaired MPC activity, and reduced both their fusion into myotubes and the formation of myofibres.

Endothelial cells regulate the structure and function of vessels and play a very important modulatory role in inflammation [26]. Preserved and efficient blood flow are also important for the migration of inflammatory cells into the muscle injury area from the bloodstream [2]. The administration of SIM in the present study did not improve blood vessel stabilization, result subsequently resulting in extensive and prolonged extravasation after myofibre injury. However, numerous experiments have confirmed a beneficial statins effect on vascular endothelial function [8, 26, 27]. The author's hypothesis investigated in the present study was that extensive and prolonged bleeding could result from the properties of SIM, such as the inhibition of platelet adhesion and aggregation at the site of vascular injury after BPVC injection, rather than from unimproved endothelial function. These antiplatelet and antithrombotic actions of statins were also confirmed in other studies [8, 11–13].

In the present study, the administration of SIM at a daily dose of 40 mg (approximately 1 mg/kg) did not increase the severity of myofibre necrosis during the first two days after muscle injury (treatment days 16 and 17). Human studies have also confirmed that SIM treatment at a daily dose of 40 mg is associated with a low risk of myopathy and rhabdomyolysis; however, a higher risk can be observed depending on the dose and time of administration [28]. In clinical trials of SIM, risk for myotoxicity risk and muscular symptoms were observed with the administration of doses of 40 or 80 mg/day for at least 3 months in approximately 18.2% of the patients [23]. In the present study, the SIM administration period was likely not long enough to directly cause muscle damage; however, starting from day 3 after the BPVC injection (treatment day 18), the severity of necrosis was significantly higher in the SIM-treated group than in the control group, and, intriguingly, this effect was accompanied by enhanced infiltration of inflammatory cells. This increased inflammation after SIM therapy that was observed starting from day 3 in the BPVC-injured muscles provided evidence that SIM did not exert an anti-inflammatory effect in the postinjury skeletal muscle regeneration. This conclusion is not in agreement with previous studies that revealed the ability of statins to decrease the recruitment of neutrophils and macrophages under many conditions [8–10, 29]. Furthermore, as observed in the SIM-treated group, a persistently high level of inflammation should accelerate the removal of cellular debris, reduce the necrosis duration, and positively affect regeneration, but these effects were not observed in the present study. The reason for this discrepancy could be associated with the extensive release of proteases and the production of cytotoxic levels of ROS by numerous neutrophils and M1 macrophages, which would contribute to more severe muscle damage [1, 2]. Moreover, the SIM-induced reduction in the phagocytic activity of inflammatory cells resulted in a slower removal of necrotic fibres, which could be another explanation for the prolonged duration of necrosis observed in the present study. This conclusion is supported by previous studies indicating that statins inhibit the secretory activity of neutrophils and macrophages and regulate the expression of factors and molecules that control their phagocytic action [10, 30, 31]. Moreover, the improper phagocytic activity of inflammatory cells detected after SIM treatment could also be the result of increased apoptosis of neutrophils and macrophages, as suggested by other investigators [9, 29] and will be the subject of further research. Furthermore, taking into account the results of the present study and the fact that M1 and M2 macrophage activity occurs starting on day 3 of the postinjury skeletal muscle regeneration, it should be assumed that SIM affects these inflammatory cells rather than neutrophils. The mechanism underlying this action and its effects on myofibre regeneration remain unelucidated. The hypothesis investigated in the current study is that SIM negatively affects phagocytic M1 rather than nonphagocytic M2 macrophages because the increased inflammatory cell numbers observed in the SIM-treated animals starting from day 3 of the experiment were correlated with increased numbers of MPCs, whereas a similar correlation was not found in the control animals. This hypothesis is also supported by the finding that M2 macrophages, which reached a peak at approximately 4 days after myofibre injury, stimulated the recruitment, proliferation, and differentiation of MPCs and promoted the repair process [1, 3].

The results strongly suggest that SIM impaired the repair phase of myofibre regeneration, as demonstrated by delayed activation of MPCs, delayed myotube formation, and delayed and decreased young myofibre formation. This finding is in agreement with previous studies that confirmed that statins can reduce the activity, growth, differentiation, and fusion of MPCs, hereby lowering the regenerative capacity of skeletal muscle [7, 17–19]. Moreover, the delayed MPC activity, as manifested by a reduction in their number, in SIM-treated animals during the first three days of the regeneration phase could be the result of the statin-mediated inhibition of MPC migration to the site of injury [1, 3]. Furthermore, the delayed myotube formation and decreased young myofibre numbers observed in this study in the SIM-treated animals could be due to the ability of statins to suppress protein and ATP synthesis and the destruction of newly formed myotubes, as has been suggested by some researchers [32, 33]. SIM could also affect the repair phase by impairing mitochondria-mediated Ca2+ homeostasis in MPCs, myotubes and young myofibres, and activating apoptosis via the mitochondrial pathway. However, these mechanisms have been investigated by other researchers, mainly through* in vitro* studies [8, 14, 33], and further* in vivo* research investigating the apoptotic action of statins in skeletal muscle regeneration is needed. Therefore, this will be the subject of further research. The reason underlying the prolongation of MPC activity after SIM administration observed in the present study remains an unanswered question. The hypothesis investigated in this study was that this effect was caused by prolonged and enhanced infiltration of inflammatory cells and necrosis in BPVC-injured muscles, which resulted in sustained stimulation of MPCs. Furthermore, excessive ROS production by neutrophils and macrophages could negatively affect the differentiation of MPCs [2].

In this study, a porcine model of skeletal muscle regeneration was used. This model was selected due to the genetic, anatomical and structural similarities between human and pig muscles [34, 35] and based on our observations of the myofibre regeneration process under different conditions in this animal model [24, 25]. Furthermore, pigs, particularly piglets, have a good and fast regenerative capacity [36]. The size, proximity, and accessibility of the left and right* longissimus lumborum* muscles in pigs were also very important criteria that were considered. The selection of this model gave us the opportunity to rapidly execute experimental procedures and allowed us to obtain a sufficient amount of muscle samples for all histological, immunohistochemical, and quantitative analyses and, most importantly, to reduce the number of animals used in the experiment. The importance of this criterion in biomedical research has been emphasized by many researchers [37, 38]. Moreover, using this large animal model, the determination of a proper dosage of SIM that could also be used in humans was much easier and suitable. For the foregoing reasons, a porcine model, similar to mouse and rats, has begun to be considered a good model for research on human skeletal muscle disorders [39].

The present study is unique because it allowed investigation of the effects of SIM on several cell populations and their interactions during postinjury skeletal muscle regeneration. The findings significantly expand the current knowledge on the deleterious effects of statins on skeletal muscle. Improved comprehension and knowledge of these mechanisms of action of SIM would have important clinical significance, particularly for risk-benefit analyses for elderly individuals who are being treated with this drug for a long period because they are potentially more vulnerable to its myotoxic side effects.

## 5. Conclusions

We demonstrated that the administration of SIM impairs postinjury skeletal myofibre regeneration. This action is multifactorial and affects both the inflammatory and repair phases, resulting in a delay in the regeneration process. In the inflammatory phase, statin increased the risk of prolonged bleeding at the site of injury and did not exert an anti-inflammatory effect but rather caused a prolonged and enhanced inflammatory response at the site of the muscle injury. Moreover, SIM appeared to reduce M1 macrophage activity, resulting in a slower removal of necrotic debris and sustained necrosis. Regarding its negative influence on the repair phase of regeneration, our findings provide evidence showing that this action is associated with the delay and prolongation of MPC activity, the delay of myotube formation, and the delay and reduction of young myofibre formation. These unique findings strengthen the available knowledge on the side effects of SIM and provide evidence showing that statin therapy is associated with an increased risk of impairment of the regenerative capacity of skeletal muscle. This new information is particularly important in relation to the extended and long-term use of SIM by elderly individuals and might be helpful for avoiding the increasing risk for age-related loss of function and the regenerative capacity of skeletal muscle during statin therapy. Considering the complexity of this action, further studies on the exact mechanisms involved in the antiregenerative action of statins on skeletal muscle are needed.

## Figures and Tables

**Table 1:** Summary of the immunohistochemical methodology.

Primary antibody	Clone	Dilution	Antigen retrieval	Visualization system
Anti–MyoD1^*∗*^	5.8A	1:50	2x3 min†, Tris-EDTA buffer pH 9.03 min	EnVision + System-HRP, Mouse (DAB)*∗*

Anti–Desmin*∗*	D33	1:50	2x3 min†, Tris-EDTA buffer pH 9.03 min	EnVision + System-HRP, Mouse (DAB)*∗*

*∗*DAKO, Glostrup, Denmark; † antigen retrieval was conducted in a microwave oven at 650 W.

**Table 2:** Scoring system used to evaluate the inflammatory and repair phases of myofibre regeneration.

Category	Evaluation criterion	Points assigned per criterion
Extravasation	Absent	0
	Focal	1
	Extensive	2

Necrosis (myofibre hypercontraction, hyalinization and fragmentation of the cytoplasm, loss of striations)	Absent	0
	Single fibres	1
	Focal	2
	Extensive	3

Inflammation (all inflammatory cells, i.e., neutrophils macrophages, lymphocytes, eosinophils)	Absent	0
	Minimal: ≤5 inflammatory cells per HPF (40x)	1
	Mild: 6-10 inflammatory cells per HPF (40x)	2
	Moderate: 11-20 inflammatory cells per HPF (40x)	3
	Marked: >20 inflammatory cells per HPF (40x)	4

Myogenic precursor cells (MPCs)	Absent	0
	Not numerous: ≤3 MPCs per HPF (40x)	1
	Moderately numerous: 4-6 MPCs per HPF (40x)	2
	Numerous: 7-12 MPCs per HPF (40x)	3
	Markedly numerous: >12 MPCs per HPF (40x)	4

Myotubes	Absent	0
	Not numerous: <3 myotubes per HPF (40x)	1
	Moderately numerous: 3-5 myotubes per HPF (40x)	2
	Numerous: >5 myotubes per HPF (40x)	3

Young myofibres	Absent	0
	Not numerous: <3 young myofibres per HPF (40x)	1
	Moderately numerous: 3-5 young myofibres per HPF (40x)	2
	Numerous: >5 young myofibres per HPF (40x)	3

## Data Availability

The data used to support the findings of this study are available from the corresponding author upon request.

## Abbreviations

BPVC: Bupivacaine hydrochloride
DAB: 3,3′-diamonobenzidine
H&E: Haematoxylin and eosin
HMG-CoA: 3-hydroxy-3-methylglutaryl-coenzyme A
MPCs: Muscle precursor cells
SIM: Simvastatin.
